# A decision analytic model to investigate the cost-effectiveness of poisoning prevention practices in households with young children

**DOI:** 10.1186/s12889-016-3334-0

**Published:** 2016-08-03

**Authors:** Felix Achana, Alex J. Sutton, Denise Kendrick, Mike Hayes, David R. Jones, Stephanie J. Hubbard, Nicola J. Cooper

**Affiliations:** 1 Clinical Trials Unit, Warwick Medical School, University of Warwick, Coventry, CV4 7AL UK; 2 Department of Health Sciences, University of Leicester, Leicester, LE1 7RH UK; 3 Division of Primary Care, University of Nottingham, Nottingham, NG7 2RD UK; 4 Child Accident Prevention Trust, London, SE16 4DG UK

**Keywords:** Economic evaluation, Public health, Injury prevention, Poisonings, Children, Decision models

## Abstract

**Background:**

Systematic reviews and a network meta-analysis show home safety education with or without the provision of safety equipment is effective in promoting poison prevention behaviours in households with children. This paper compares the cost-effectiveness of home safety interventions to promote poison prevention practices.

**Methods:**

A probabilistic decision-analytic model simulates healthcare costs and benefits for a hypothetical cohort of under 5 year olds. The model compares the cost-effectiveness of home safety education, home safety inspections, provision of free or low cost safety equipment and fitting of equipment. Analyses are conducted from a UK National Health Service and Personal Social Services perspective and expressed in 2012 prices.

**Results:**

Education without safety inspection, provision or fitting of equipment was the most cost-effective strategy for promoting safe storage of medicines with an incremental cost-effectiveness ratio of £2888 (95 % credible interval (CrI) £1990–£5774) per poison case avoided or £41,330 (95%CrI £20,007–£91,534) per QALY gained compared with usual care. Compared to usual care, home safety interventions were not cost-effective in promoting safe storage of other household products.

**Conclusion:**

Education offers better value for money than more intensive but expensive strategies for preventing medicinal poisonings, but is only likely to be cost-effective at £30,000 per QALY gained for families in disadvantaged areas and for those with more than one child. There was considerable uncertainty in cost-effectiveness estimates due to paucity of evidence on model parameters. Policy makers should consider both costs and effectiveness of competing interventions to ensure efficient use of resources.

**Electronic supplementary material:**

The online version of this article (doi:10.1186/s12889-016-3334-0) contains supplementary material, which is available to authorized users.

## Background

Globally poisonings account for approximately 45,000 deaths [1] and approximately 2.4 million disability adjusted life years (DALYS) lost [2] each year in children and young people aged 0–19 years. Poisonings among 0–15 year olds have been estimated to cost the NHS more than £2 million each year [3]. In the USA, despite an estimated saving of $7–$15 for every $1 spent on poison control centres [4], non-fatal poisonings resulted in $48 million medical costs for hospitalisations and Emergency Department (ED) attendances in 2005 for the under 5 s [5].

Systematic reviews [6–8] and a recent network meta-analysis [9] show home safety interventions including education, home safety inspections, provision of free or low cost safety equipment and fitting of equipment are effective in promoting some poison prevention practices in families with children. However there is little published evidence on the cost-effectiveness of these interventions. This paper builds on the findings from the network meta-analysis and presents a decision analytic model to investigate the cost effectiveness of poison prevention practices in households with children aged 0–4 years.

We consider strategies that promote safe storage of medicines separately from those that promote safe storage of other household products. We customised our model for i) under 5 year olds from socio-economic disadvantaged groups^1^ whom the evidence suggest are at increased risk of unintentional injury compared to those from a well-off family background [10], and ii) households with multiple children as the benefit of the intervention are likely to be greater than for families with a single child.^2^ Finally, as a way of improving transparency of the modelling exercise, and prior to presenting a cost-utility analysis, we first present a cost-effectiveness analysis in which the health benefits are expressed in numbers of poison cases avoided, the natural units of the economic evaluation displayed for each intervention cohort.

## Methods

### Modelling framework

We developed a probabilistic decision model to compare the cost-effectiveness of seven home safety interventions to increase uptake of poison prevention behaviours in households with children aged 0–4 years. The intervention strategies evaluated in the model were identified from a recently published systematic review and network meta-analysis [9] and include:

1. Usual care
2. Education (more than usual safety education)
3. Provision of low cost/free equipment
4. Education + provision of low cost/free equipment
5. Education + provision of low cost/free equipment + home safety inspection
6. Education + provision of low cost/free equipment + fitting (i.e. free installation of equipment)
7. Education + provision of low cost/free equipment + home safety inspection + fitting

We categorised the intervention strategies into these single and multi-component treatment packages based on the information reported in the individual study reports. The control intervention from individual studies was classed as usual care if the study reported the control group as ‘usual safety education’, ‘standard safety practice or advice’ or ‘no safety education’ (i.e. no or do-nothing intervention control groups). Education was taken to mean that provided in addition to usual or standard safety education delivered by face-to-face contact with a trained health professional or by an educational leaflet. Free or low cost safety equipment included the provision of poison-related equipment such as cupboard or drawer locks; some interventions also provided other home safety equipment not aimed at poison prevention (e.g. smoke alarms, safety gates etc). Home safety inspection refers to home visits including inspections carried out by trained health and other professionals. Finally “fitting” refers to installation of safety equipment by a trained professional.

We constructed a cost-utility model to estimate mean costs and Quality Adjusted Life Years (QALYs) associated with the 7 strategies over a life time horizon (assumed to be 100 years). We also performed a cost-effectiveness analysis to estimate costs and consequences in the form of number of poison cases associated with each intervention over the first 5 years of life. Further technical details of the model structure and parameterisation are given in Achana [11] PhD Thesis. Separate analyses investigated the cost-effectiveness of interventions to promote poison prevention practices for medicinal poisonings (ICD-10 codes X40-X44) and non-medicinal (other household products related) poisonings (ICD-10 codes X45-X49).

Table 1 summarises the key features of the base case analysis. The modelled population is children aged 0–4 years, the exposure variable is safe storage of medicines or of other household products, the comparator is usual care and the outcome variable is medically-attended unintentional actual or suspected poisoning. Safe storage is defined as storage above adult eye level or in locked cabinets and/or drawers so that it is out of reach of children [7]. The unit of analysis is the household when estimating the relative effectiveness of interventions but the individual when modelling cost-effectiveness. Households were chosen as the unit of analysis in the intervention model because households are the level at which interventions are most usually provided. It is assumed that interventions act to increase the proportion of households with safe storage of poisons (at a rate determined by the relative effectiveness estimates) above and beyond the baseline prevalence of safety practices. Households with safe storage are assumed to present a lower risk of unintentional ingestion compared to households without safe storage. The aim of the decision analysis is to estimate the likelihood of an unintentional poisoning event in pre-school children for a given intervention and use this to estimate costs and consequences/benefits associated with treating such events over the lifetime of the individual. The cost-effectiveness of home safety interventions will be determined with reference to the NICE cost-effectiveness thresholds of between £20,000 and £30,000 per additional QALY, where an ICER below £20,000 per QALY is generally considered to be cost-effective [12].

**Table 1:** Base case analyses

Parameter	Description
Type of economic evaluation	Cost-effectiveness and cost-utility analysis
Modelled population	Preschool children (0–4 years of age)
Exposure variables	1) Safe storage of medicines 2) Safe storage of other household products
Outcome event	Unintentional ingestion of potential toxic substance
Unit of analysis	Household with one child
Perspective on costs	UK NHS and Personal and Social Services (PSS)
Health outcomes (Utilities)	Quality Adjusted-Life Year (QALY)
Base year for calculating costs/prices	2012
Currency unit	British pound (£)
Hypothetical cohort size	100,000 households
Effectiveness evidence	Network meta-analyses (Achana et al 2015) [9]
Comparator or reference intervention	Usual care intervention
Number of intervention strategies	7
Number of health states (Markov model)	6
Cycle length for Markov model	1 year
Half-cycle correction	No
Time horizon	100 years
Discount rate for costs	3.5 %
Discount rate for utilities	3.5 %

### Model structure

**Fig. 1:**
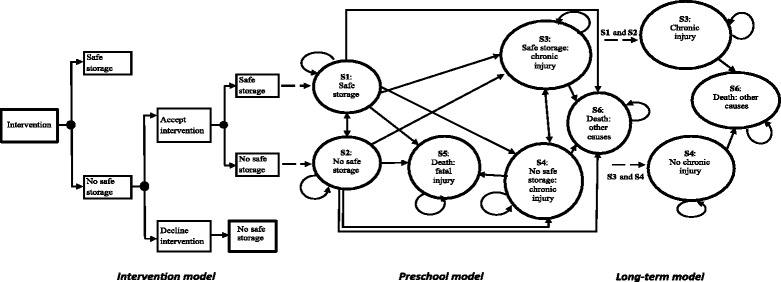
Decision model structure. Arrow heads indicate direction of movement of households/individuals through the model

A cohort simulation model is developed to estimate healthcare utilisation costs and health outcomes (number of poison cases in the cost-effectiveness analysis and QALYs in the cost-utility analysis) associated with home safety intervention compared to usual care intervention. The model consists of a decision tree and Markov model structures and is based on two previous decision analytic models that investigated the cost-effectiveness of smoke alarm give-away schemes on health outcomes in children [13, 14]. Figure 1 shows the structure of the model with three distinct but interlinked sub-models:

**Fig. 2:**
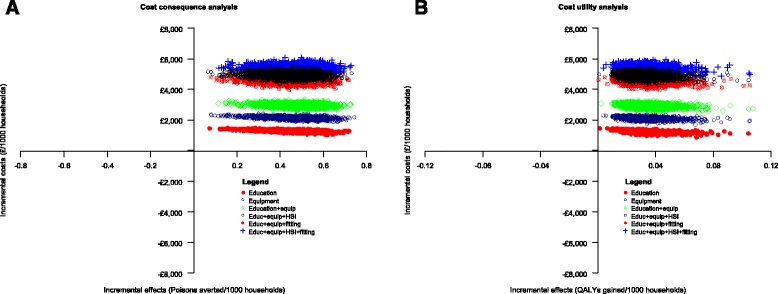
Cost-effectiveness plane displaying simulated ICERs for each home safety intervention compared to usual care (Medicinal poison prevention model). Plot A refers to the cost-effectiveness analysis and plot B to the cost-utility analysis

**Fig. 3:**
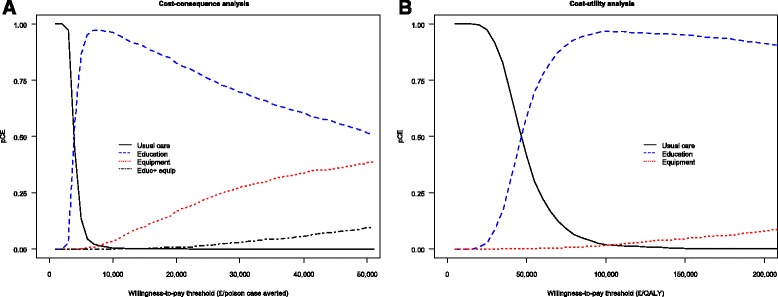
Cost-effectiveness acceptability curves for base case analysis Curves indicate the probability that each intervention is the most cos-effective for a range of willingness-to-pay threshold (Medicinal poison prevention model). Plot A refers to cost-effectiveness analysis and B to cost-utility analysis. Only interventions with a non-zero probability of being cost-effective are displayed

i) a first stage decision tree model referred to as the ‘intervention model’,
ii) a second stage Markov state transition model referred to as the ‘preschool model’ and
iii) a third stage Markov state transition model referred to as the ‘long-term model’.

#### Stage 1: Intervention model

In this part of the model, a decision tree is used to estimate costs and outcomes associated with the interventions being evaluated. The intervention model accounts for the baseline prevalence of safety practices in the general population, the acceptance rate of interventions in the modelled population, and the relative effectiveness of interventions in promoting uptake of poison prevention practices in the home. Costs incurred in this part of the model are costs associated with providing the intervention; a detailed description of how these are estimated is provided in the data sources section. Health outcomes in the intervention model are the number of households with and without safe storage over in the first cycle of the model. The outcomes from the intervention model serve as inputs for the second stage ‘preschool’ model which is described below.

#### Stage 2: Preschool model

The preschool model uses a Markov structure to estimate the costs and QALYs associated with each intervention strategy being evaluated in the first 5 years of life (ages 0–4 years). There are six distinct health states (Fig. 1): safe storage (S1), no safe storage (S2), safe storage with chronic injury (S3), no safe storage with chronic injury (S4), death from poisoning injury (S5) and death from causes unrelated to poisoning injury (S6). All individuals initially enter the preschool model in either the safe storage (S1) or the no safe storage (S2) states as determined by the intervention model. The transition probabilities that govern the movement of individuals between health states are presented in Table 2 for medicinal poisonings model and Table 3 for non-medicinal (other household products) poisonings model. These were estimated based on evidence from the literature. The two death states, S5 and S6, are absorbing states from which individuals cannot leave or move to another state in subsequent cycles of the model.

**Table 2:** Probabilities used in the medicinal poisonings model

Parameter	Description	Data sources	Derivation of required probabilities	Point estimate (standard error (SE)	Distribution
*pSafe*	Baseline prevalence of safe storage of medicines	Case control study of risk and protective factors for poison injuries in under 5 year olds (Kendrick et al, paper om press).	Number of households with safe storage of medicines/Total number of households selected as community controls in case control of risk and protective factors in children under 5 years old (Kendrick et al, paper om press).	1527/2033 = 0.75	Beta
*pAccept*	Probability of accepting the intervention	Saramago, Cooper et al., 2015 [14]	Assumption based on value in Functional smoke alarm model. Assumed the same for all interventions.	0.90	Fixed
*pEff*	Probability of safe storage of medicines given the intervention	Achana et al., 2015 [9]	(1) Usual care (2) Education (3) Provision of low cost/free low cost/free equipment (4) Education + provision of low cost/free equipment (5) Education + equipment + home safety inspection (6) Education + equipment + fitting (7) Education + equipment + home safety inspection + fitting	0.87 (95%CrI 0.56, 0.98) 0.90 (95%CrI 0.61, 0.98) 0.93 (95%CrI 0.65, 0.99) 0.94 (95%CrI 0.74, 0.99) 0.90 (95%CrI 0.56, 0.99) 0.90 (95%CrI 0.59, 0.98) 0.93 (95%CrI 0.66, 0.99)	Posterior distribution of absolute intervention effects from network meta-analysis.
*pIngest*	Probability of unintentional exposure/ingestion	Orton et al., 2014 [10] Tyrrell et al., 2012 [33] Office for National Statistics, 2013 [34]	Orton et al [10] – incidence of poisonings in the UK for period 2005–2009 is 30.1 (95 % CI 29.1–31.2). Mid-year population estimates of under 5 year olds in the UK in 2012 = 3996400 [34]. Hence number of poisoning cases = (30.1*3996400)/10000 = 12029. The numerator (n = 12029*0.6 = 7217) was derived based on information in [33] suggesting that 1316 (60 %) of the 2193 medically reported poisonings identified in the THIN database were due to ingestion of a medicinal substance.	7217/3996400 = 0.001806	Beta
*orIngest*	Relative risk of exposure to a medicinal substance comparing children with a poisoning to community controls.	Case control study of risk and protective factors for poison injuries in under 5 year olds (unpublished study)	Community controlled adjusted analysis odds ratio for safe storage versus no safe storage = 1.83 (95 % CI 1.38–2.42)	Log odds ratio (SE) = -0.604 (0.143)	Normal
*pAmb*	Probability of using emergency ambulance.	Hospital Episode Statistics, 2012	Hospital Episode Statistics (2012b): 24.2 % of all cases arrived by emergency transfer (ambulance/helicopter).	0.242	Fixed
*pAdmit*	Probability of in-patient admission following a medicinal poisoning injury (ICD-10: X40-X44).	Hospital Episode Statistics, 2013 [35] Office for National Statistics, 2013 [34]	Hospital Episode Statistics, 2012–2013) [35]: Number of poisoning cases (X40-X44) admitted in 0–4 years olds (period 2012–2013) in England = 3909. Scaled up by a factor of 1.163 (i.e. 3909*1.163 = 4546 cases for whole of UK) based on mid-2012 population estimates for UK and England [34].	4546/7217 = 0.63	Beta
*pSevere*	Probability of severe poisoning injury	Mowry et al., 2013 [36]	NPDS 2012 report [36], Table 13, page 968) 1.91 % of major poisoning cases (across all age groups) resulted in a chronic health condition. Numerator = 0.019* 4546 = 87.	87/4546 = 0.0191	Beta
*pFatal*	Probability of fatal poisoning injury.	Office for National Statistics, 2012 [37]	UK mortality statistics [37]. 1 fatality from medicinal poisonings in 0–4 years old (assumed fatality occur after a long inpatient stay).	1/86 = 0.0116	Beta
*pDead*	UK mortality statistics	Office for National Statistics, 2010 [38]	UK mortality statistics [38]		Normal

**Table 3:** Probabilities used to inform none-medicinal (other household products) decision model

Parameter	Description	Data sources	Derivation of required probabilities	Point estimate (standard error (SE)	Distribution
*pSafe*	Baseline prevalence of safe storage of non-medicines	Kendrick et al (paper om press)	Number of households with safe other household products/Total number of households selected as community controls in case control of risk and protective factors in children under 5 years old (Kendrick et al, paper om press).	948/1138 = 0.83	Beta
*pAccept*	Probability of accepting the intervention	Saramago, Cooper et al., 2015 [14]	Assumption based on value in Functional smoke alarm model [14]. Assumed the same for all interventions.	0.90	Fixed
*pEff*	Probability of safe storage of medicines given the intervention	Achana et al., 2015 [9]	(1) Usual care (2) Education (3) Provision of low cost/free low cost/free equipment (4) Education + provision of low cost/free equipment (5) Education + equipment + home safety inspection (6) Education + equipment + fitting (7) Education + equipment + home safety inspection + fitting	0.62 (95%CrI 0.34–0.81) 0.66 (95%CrI 0.38–0.87) 0.36 (95%CrI 0.00–1.00) 0.78 (95%CrI 0.48–0.94) 0.80 (95%CrI 0.51–0.94) 0.68 (95%CrI 0.32–0.90) 0.50 (95%CrI 0.00–1.00)	Posterior distribution of absolute intervention effects from network meta-analysis.
*pIngest*	Probability of unintentional exposure/ingestion	Orton et al., 2014 [10] Tyrrell et al., 2012 [33] Office for National Statistics, 2013 [34]	Number of unintentional poisoning cases among children under 5 years in 2012 = 12029 (see Table 2 above). Forty percent of poisoning cases is due to ingestion of non-medicinal substance [33]. Hence numerator = 12029*.4 = 4812.	4812/3996400 = 0.001204	Beta
*orIngest*	Relative risk of exposure to a medicinal substance comparing children with a poisoning to community controls.	Case control study of risk and protective factors for poison injuries in under 5 year olds (unpublished study)	Community controlled adjusted analysis odds ratio for safe storage versus no safe storage = 0.77 (95 % CI 0.59–0.99)	Log odds ratio (SE) = 0.2614 (0.132)	Normal
*pAmb*	Probability of using emergency ambulance.	Hospital Episode Statistics, 2012	Hospital Episode Statistics (2012b): 24.2 % of all cases arrived by emergency transfer (ambulance/helicopter).	0.242	Fixed
*pAdmit*	Probability of in-patient admission following a medicinal poisoning injury (ICD-10: X40-X44).	Hospital Episode Statistics, 2013 [35] Office for National Statistics, 2013 [34]	Hospital Episode Statistics, 2012–2013) [35]: Number of poisoning cases (X45–X49) admitted in 0-4 years olds (period 2012–2013) in England = 1377. Scaled up by a factor of 1.163 (i.e. 1377*1.16 = 1597 cases for whole of UK) based on mid-2012 population estimates for UK and England, ONS 2012a [34].	1597/4812 = 0.3318	Beta
*pSevere*	Probability of severe poisoning injury	Mowry et al., 2013 [36]	NPDS 2012 report [36], Table 13, page 968) 1.91 % of major poisoning cases (across all age groups) resulted in a chronic health condition. Numerator = 0.019* 1597 = 30.	30/1597 = 0.0191	Beta
*pFatal*	Probability of fatal poisoning injury.	Office for National Statistics, 2012 [37]	UK mortality statistics [37]. 1 fatality from medicinal poisonings in 0–4 years old (assumed fatality occur after a long inpatient stay).	1/30 = 0.033	Beta
*pDead*	UK mortality statistics	Office for National Statistics, 2010 [38]	UK mortality statistics [38]		Normal

#### Stage 3: Long-term model

This part of the model applies to individuals aged 5 years and older. It uses a 3-state Markov structure to quantify lifetime costs and QALYs associated with chronic/long term health conditions resulting from unintentional poisonings in the 5 years (Fig. 1). No cases of unintentional poisonings are assumed to occur during this period (i.e. at ages above 5 years; or if they do occur they are not taken into account in the model).

### Implementation

The model is implemented within a Bayesian framework utilising the comprehensive decision modelling approach [15, 16]. All data sources used to inform model parameters are reported in Tables 1, 2, 3, 4 and 5 and described below. Parameters are estimated by means of Markov Chain Monte Carlo (MCMC) simulations implemented in the WinBUGS software. Estimates were obtained after running three MCMC chains for 30 000 iterations using disparate starting values. The first 10,000 iterations from each chain were discarded as ‘burn-in’ samples to ensure that the starting values do not influence the samples on which inference is based [17]. The WinBUGS code used to implement the model is available from the authors. Uncertainty in model parameters was included by assigning probability distributions to parameters and also through deterministic sensitivity and scenario analyses. A list of model assumption is presented in Additional file 1 and the sensitivity analyses that were conducted are displayed in Additional file 2.

**Table 4:** Interventions and healthcare costs

Parameter	Description	Data sources	Derivation of required cost information	Point estimate (standard error (SE))	Distribution
Intervention costs^a^					
*cFixed*	Fixed cost of setting up an intervention scheme for 100 000 households	Saramago et al. 2014 [14]	Saramarago Cooper et al. [14]	£79,529	Fixed
*cAccept*	Cost of accepting intervention		Assumption	£0.40	Fixed
*cTravel*	Travel time and costs		Travel time and costs Nottingham home safety equipment scheme^b^ hourly rate including on costs and vehicle costs is £25 (estimate obtained through personal communication) to install 5 items of safety equipment. We allocated 1/5 th of hourly rate to poisoning prevention activities.	£5.00	Fixed
Intervention schemes					
*cInter1*	Usual care		No cost associated with usual care as no intervention is provided.	£0.00	Fixed
*cInter2*	Education		Estimate based on 5 min of health visitors time costing £44 (interquartile rage £33–£54) [19]. This assumes education is provided during contact for other reasons (e.g. child health surveillance contact). No travel costs are included as it is assumed contact either occurs in clinic or during home visit for other reasons.	£3.67	
*cInter3*	Free or low cost safety equipment.		2x kitchen cabinet locks (based on providing one pop-It Lock™ costing £2.65 and one magnetic lock costing £4.80 each plus vat). This assumes locks are provided during contact for other reasons (e.g. child health surveillance contact). No travel costs are included as it is assumed contact either occurs in clinic or during home visit for other reasons.	£7.45	Fixed
*cInter4*	Education + equipment		Cost of education and two cabinet locks = £3.76 + £7.45. This assumes education and locks are provided during contact for other reasons (e.g. child health surveillance contact). No travel costs are included as it is assumed contact either occurs in clinic or during home visit for other reasons.	£11.12	Fixed
*cInter5*	Education + equipment + home safety inspection		Cost of education, two cabinet locks, home safety inspection and travel = £3.67 + £7.45 + £3.67 + £4.99. This assumes education, home safety inspection and locks are provided by a health visitor during contact covering prevention of a range of injuries, of which poison prevention is a part. It assumes poison prevention education and home safety inspection each takes 5 min of health visitor time.	£19.78	Fixed
*cInter6*	Education + equipment + fitting		Education + equipment + fitting Cost of education, two cabinet locks and fitting of two locks (assumed takes 5 min of safety equipment fitters time at £25 per hour) and travel = £3.67 + £7.45 + £2.08 + £4.99. This assumes education is provided by a health visitor during a contact for other reasons (e.g. child health surveillance) and locks and fitting of locks are provided by a safety equipment scheme during contact covering prevention of a range of injuries, of which poison prevention is a part. It assumes poison prevention education takes 5 min of health visitor time and fitting of locks takes 5 min of safety equipment fitters’ time at £25 per hour.	£18.19	Fixed
*cInter7*	Education + equipment + home inspection + fitting		Education + equipment + home inspection + fitting Cost of education, two cabinet locks, home inspection, fitting of locks and travel = £3.67 + £7.45 + £3.67 + £2.08 + £4.99. This assumes education is provided by a health visitor during a contact for other reasons (e.g. child health surveillance) and home safety inspection, locks and fitting of locks are provided by a safety equipment scheme during contact covering prevention of a range of injuries, of which poison prevention is a part. It assumes poiso n prevention education takes 5 min of health visitor time and the home safety inspection and fitting of locks each take 5 min of safety equipment fitters’ time at £25 per hour.	£21.86	Fixed
Healthcare costs (Hospital and primary care costs)					
*cAmb*	Cost of emergency transfers	PSSRU [19]		£263 (SE = £21.48)	Gamma
*cED1*	Cost of emergency department treatment of cases not leading to hospital inpatient stay (minor injury)	PSSRU [19]		£112 (SE = £27.41)	Gamma
*cED2*	Cost of emergency department treatment for cases leading to hospital inpatient stay (major injury)	PSSRU [19]		£146 (SE = £42.22)	Gamma
*cAdmit1*	Cost of a non-elective short (<2 days) inpatient admission	PSSRU [19]		£586 (SE = £223.70)	Gamma
*cAdmit2*	Cost of a non-elective long (≥2 days) inpatient admission	PSSRU [19]		£2461 (SE = £810.37)	Gamma
*cChro*	Annual cost of chronic ill-health	HALO study (Nicholl et al.: The Long Term Health and Healthcare Outcomes of Accidental Injury (The HALO Study) Final Report, (Unpublished))		£386.42 (SE = £96.72)	Gamma
*cFatal*	Cost of fatal injury	Saramago et al., 2014 [14]		£205.50	Fixed
*cGP*	Cost of 11.7 min GP consultation	PSSRU [19]		£43	Fixed

^a^ In the base case analysis, assumed that a specified amount of a home visitors time is spent on the poisoning prevention part of the visit. Hence we assumed 5 min of home visitors time is allocated for poisoning education, 5 min of safety fitter’s time for home safety inspection relevant to poisoning prevention and 5 min of safety fitter’s time for fitting two cupboard locks

^b^ The Nottingham safety equipment scheme provides a maximum number of items to be installed 2 gates, 2 cupboard catches, window catches, blind chord clips and a bath mat. Therefore assume 1/5th of travel time and costs are for poisoning prevention

**Table 5:** Utilities (quality adjusted life years) used in the analysis (both medicinal and non-medicinal poisoning models)

Parameter	Description	Source	Point estimate (standard error)	Distribution
*uPop*	UK non-injured population utilities	[21]	<25 years 0.94 (SE = 0.12) 25–34 years 0.93 (SE = 0.15) 35–44 years 0.91 (SE = 0.16) 45–54 years 0.85 (SE = 0.25) 55–54 years 0.80 (SE = 0.26) 65–74 years 0.78 (SE = 0.26) ≥75 years 0.73 (SE = 0.27)	Normal
*uMinor*	Utility deficit for minor injury	Miller 2000 [23]. Assumed standard error is 10 % of mean [24, 39]	0.03 (SE = 0.003)	Beta
*uModerate*	Utility deficit for moderate injury	Utility decrement 0.046 for poisoning injury [22]	0.046 (SE = 0.0046)	Beta
*uSevere*	Utility deficit for severe injury	Utility decrement 0.046 for poisoning injury [22] and decrement associated with injury of 0.1 from the HALO study	0.146 (SE = 0.0146)	Beta
*uChronic*	Utility deficit associated with chronic injury per year	HALO Study (Nicholl et al. The Long Term Health and Healthcare Outcomes of Accidental Injury (The HALO Study) Final Report, (Unpublished))	0.10 (SE = 0.025)	Beta

## Data sources

### Probabilities

Tables 2 and 3 summarise the sources of data used to inform the model for storage of medicines and safe storage of other household products respectively. These include the baseline prevalence of safety practices in the general population, the acceptance rate of the interventions, the probability that the intervention is effective in promoting safe storage, the probability of unintentional poisonings given no safe storage, the relative risk of unintentional poisonings given safe storage, probability of using an emergency ambulance, the probability of inpatient admission, and the probability of minor, moderate, severe and fatal injury.

### Costs

Table 4 provides details of resource use and costs used in the model. Costs are considered from the UK National Health Service and Personal Social Services (NHS/PSS) perspective; therefore only the cost of providing the interventions and the NHS costs of treating unintentional poisoning related injuries are considered. Costs incurred prior to 2012 are converted to 2012 prices using the Bank of England inflation calculator [18].

#### Intervention costs

Intervention costs were estimated at the household level as a sum of the costs of resource use associated with providing the constituent components of the intervention. A detailed description of the methods and the assumptions that were made in deriving the intervention costs is presented in Table 4. For example, the cost of home safety education as a standalone intervention was estimated to be £3.67 (in 2012 prices) based on 5 min of health visitors time costing £44 per hour according to the unit costs of health and social care 2012 [19]. No travel time was added as it was assumed that contact either occurred in the clinic or that during a home visit for another reasons, for example, health visitation by a midwife health visitor. If the intervention had education and home safety inspection components, these were assumed to be provided by a health visitor during contact covering prevention of a range of injuries, of which poison prevention is a part. It assumes poison prevention education and home safety inspection each takes 5 min of a health visitor’s time at £44 per hour [19]. We assumed that two cupboard locks (1 pop-It Lock™ costing £2.65 and 1 magnetic lock costing £4.80 each) are provided as part of an equipment provision scheme. Fitting of the two cupboard locks was assumed to take 5 min of an equipment fitter’s time estimated at £25 per hour. For interventions that require the health visitor or equipment fitter to travel to family homes, we used estimates of travel costs from Nottingham home safety equipment scheme based on an hourly rate of £25 in 2012 prices (estimate obtained through personal communication) and assigned 20 % of this to poison prevention related activities and advice. Based on the above assumptions, cost assigned to each intervention were as follows:

1. Usual care (£0.00)
2. Education (£3.67)
3. Provision of free or low costs equipment (two locks = £7.45)
4. Education + equipment (£11.12)
5. Education + equipment + home safety inspection (£19.78)
6. Education + equipment + fitting (£18.19) and
7. Education + equipment + home safety inspection + fitting (£21.86).

#### Healthcare utilisation/treatment costs

Table 4 provides details of healthcare resource use and costs used in the model. The cost of treating unintentional poisoning injury was estimated based on NHS reference costs for hospital services obtained from PSSRU Unit Costs of Health and Social Care [19]. In the model, it was assumed that all medically reported cases of unintentional poisoning are taken to the emergency department for initial assessment and or treatment. In the emergency department, cases are triaged as minor (requiring no inpatient stay), moderate (requiring short inpatient admission) or severe (requiring long inpatient admission). The costs of a minor injury were estimated as £175.12 based on the mean cost of an emergency ambulance (£263), weighted by the proportion (0.24) of cases arriving at emergency departments by ambulance across England in 2011-12 [20] and emergency department costs for cases not admitted (£112). Similarly, the costs of treating a moderate injury were estimated as £795.12 based on a weighted cost of emergency ambulance (£63.12), mean costs of emergency department treatment for cases leading to inpatient admission (£146) and mean cost of non-elective short inpatient stay (£586). The cost of severe injury was estimated as £2670 obtained by replacing the mean cost of a short inpatient stay used for moderate injuries to a mean cost of non-elective long inpatient stay (£2461). Other health sector costs considered in the analysis were the additional costs of a poisoning related fatality (i.e. coroners, autopsy), follow-up GP consultation lasting 11 min, health visitors’ time lasting 40 min and the annual costs of chronic ill-health obtained from the HALO study report (Nicholl et al.: The Long Term Health and Healthcare Outcomes of Accidental Injury (The HALO Study) Final Report, (Unpublished)).

### Utilities

The unit of health benefit (utility) in the cost-analysis is the quality adjusted life-year (QALY). Baseline utilities for non-injured individuals (i.e. those with no poison-related injury) were taken from general UK population utility norms [21] for people aged 18 years and above (Table 5). We assumed that individuals younger than 18 years had the same utility tariffs as the 18–25 year age range in Kind et al. [21]. Utility decrements associated with poisoning related injury were obtained from two American studies [22, 23]. Miller et al. [23] reported a QALY loss of 0.03 while Miller et al. [22] reported a QALY loss of 0.046 for childhood poisoning injury (Table 5). These two figures are estimates of the utility for any poison-related injury irrespective of the severity of the injury. We assumed that minor poisoning injury were associated with the 0.03 QALY decrement (the lower of the two estimates) and moderate injury was associated with QALY decrement of 0.046 (the upper estimate). The utility decrement for severe injury was obtained by adding the upper estimate of 0.046 to a QALY decrement of 0.10 associated with the chronic injury state in the HALO study (Nicholl et al.: The Long Term Health and Healthcare Outcomes of Accidental Injury (The HALO Study) Final Report, (Unpublished)). Uncertainty around the mean utility tariffs was incorporated by assuming that the standard error of each utility decrement equals 10 % of the mean value [24]. We investigated the impact of these assumptions through sensitivity analysis.

## Results

### Medicinal poisoning base case

The results of the base case cost-effectiveness analysis for safe storage of medicines are presented in Table 6. The incremental cost-effectiveness ratios (ICERs) per poison avoided increased with increasing intensity of the intervention. Compared with usual care, the intervention with the lowest ICER was education with an ICER of £2888 (95 % CrI £1990–£5774), followed by provision of safety equipment (one each of 2 types of cupboard/drawer lock) with an ICER of £4553 (95 % CrI £3284–£8892) and education plus equipment with an ICER of £6195 (95%CrI £4519–£11,030). The results of the base case cost-utility analysis for safe storage of medicines are presented in Table 7. The average utility (i.e. health benefit) accumulated over the life time horizon (assumed to be 100 years) was about 25056 QALYs for 1000 individuals or slightly more than 25 QALYs over the life-time of an individual. Usual care was estimated to have the lowest cost per 1000 households (mean £4169; 95 % CrI £2872–£6045) whilst the most intensive intervention consisting of ‘education, home inspection, provision of low cost or free equipment and fittings’ had the highest costs (mean £9506, 95 % CrI £8166–£11,410). Compared to usual care, the incremental cost-effectiveness ratio was lowest for education (mean ICER = £41,330; 95 % CrI £20,007–£91,534 per QALY gained). The ICER for education plus equipment compared with usual care was £90,615 (95 % CrI £46,258–£182,517). Provision of low costs equipment provided the same QALY gain as education for a higher costs, hence was dominated by education in health economic terms. Education plus equipment and home safety inspection, education plus equipment and fitting, and education plus equipment plus home safety inspection and fitting were all associated with higher costs than more effective interventions and so were also dominated. Education was the most cost-effective intervention with probability of 0.81 followed by provision of low cost or free equipment if the cost-effectiveness threshold is £30,000 per poison case avoided.

Figure 2 is a cost-effectiveness plane showing 4000 simulated ICER samples for each of the six interventions compared to usual care in the cost-effectiveness analysis (plot A) and cost-utility analysis (plot B). All ICERs lie in the north-east quadrant of the plane, suggesting that the interventions were more costly but also more effective than usual care. Figure 3 is a plot of the probability that each intervention is the most cost-effective for a range of willingness-to-pay thresholds. The plot shows that compared to usual care, home safety interventions are only cost-effective only if the willingness-to-pay threshold is about £3,000 per poison avoided (plot A) and above 50,000 per QALY gained (plot B).

**Table 6:** Base case cost-effectiveness estimates for medicinal poisonings (cost-effectiveness) model

Intervention	Expected benefits (Poison cases)^a^	Expected Costs (£)^a^	Incremental benefits (Poisoning avoided) ^a^	Incremental Costs (£)^a^	ICER (£/Poisoning avoided)	Probability intervention is cost-effective at £30,000/QALY	Probability intervention is cost-effective at £50,000/QALY
UC	5.622 (4.988, 6.362)	3617 (2372, 5398)				0.000	0.000
E	5.163 (4.422, 6.073)	4937 (3737, 6606)	0.453 (0.246, 0.617)	1316 (1165, 1424)	2888 (1990, 5774)	0.811	0.6885
FE	5.142 (4.398, 6.071)	5777 (4554, 7452)	0.472 (0.251, 0.632)	2155 (1969, 2307)	4553 (3284, 8892)	0.181	0.279
E + FE	5.137 (4.398, 6.056)	6587 (5352, 8254)	0.479 (0.274, 0.64)	2973 (2749, 3172)	6195 (4519, 11030)	0.007	0.0315
E + FE + HSI	5.176 (4.429, 6.105)	8541 (7248, 10250)	0.443 (0.219, 0.616)	4926 (4576, 5261)	Dominated	0.000	0.000
E + FE + F	5.168 (4.421, 6.076)	8178 (6899, 9895)	0.449 (0.24, 0.627)	4566 (4243, 4878)	Dominated	0.000	0.000
E + FE + HSI + F	5.148 (4.401, 6.071)	8998 (7691, 10740)	0.468 (0.259, 0.634)	5382 (4999, 5747)	Dominated	0.000	0.000

^a^ Figures are expected benefits (95 % credibility interval) and expected costs (95 % credibility interval) per 1000 households over a 5 year time horizon

Interventions

*UC* usual care

*E* education

*FE* provision of low cost/free equipment

*E + FE* education + provision of low cost/free equipment

*E + FE + HSI* education + provision of low cost/free equipment + home safety inspection

*E+ FE + F* education + provision of low cost/free equipment + Fitting

*E + FE + HSI + F+ fitting* education + provision of low cost/free equipment + home safety inspection + Fitting

**Table 7:** Base case cost-effectiveness estimates medicinal poisons (cost-utility) model

Intervention	Expected benefits (QALY)^a^	Expected Costs (£)^a^	Incremental benefits (QALY)^a^	Incremental Costs (£)^a^	ICER (£/QALY)	Probability intervention is cost-effective at £30,000/QALY	Probability intervention is cost-effective at £50,000/QALY
UC	25056.559 (25039.293, 25073.828)	4169 (2872, 6045)				0.828	0.301
E	25056.578 (25039.328, 5073.855)	5435 (4197, 7271)	0.031 (0.015, 0.059)	1273 (1110, 1398)	41330 (20007, 91534)	0.172	0.698
FE	25056.578 (25039.322, 5073.855)	6270 (5027, 8099)	0.031 (0.016, 0.061)	2111 (1926, 2275)	Dominated	0.000	0.002
E + FE	25056.578 (25039.328, 5073.857)	7089 (5829, 8921)	0.032 (0.017, 0.062)	2927 (2701, 3132)	90615 (46258, 184517)	0.000	0.000
E + FE + HSI	25056.578 (25039.326, 5073.857)	9051 (7737, 10930)	0.030 (0.015, 0.059)	4881 (4541, 5227)	Dominated	0.000	0.000
E + FE + F	25056.578 (25039.326, 5073.855)	8695 (7392, 10570)	0.030 (0.015, 0.06)	4522 (4209, 4844)	Dominated	0.000	0.000
E + FE + HSI + F	25056.580 (25039.328, 5073.857)	9506 (8166, 11410)	0.031 (0.016, 0.061)	5338 (4954, 5717)	Dominated	0.000	0.000

^a^ Figures are expected QALY (95 % credibility interval) and expected costs (95 % credibility interval) per 1000 households over lifetime horizon (assumed 100 years)

*ICER* incremental cost-effectiveness ratio

*QALYs* quality-adjusted life years

Interventions

*UC* usual care

*E* education

*FE* provision of low cost/free equipment

*E + FE* education + provision of low cost/free equipment

*E + FE + HSI* education + provision of low cost/free equipment + home safety inspection

*E+ FE + F* education + provision of low cost/free equipment + Fitting

*E + FE + HSI + F+ fitting* education + provision of low cost/free equipment + home safety inspection + Fitting

### Medicinal poisonings sensitivity analyses

Table 8 displays the results of sensitivity analyses for the medicinal poisonings decision model. Sensitivity analyses for the non-medicinal poisonings model are presented in the accompanying supplementary material. Only the cost-utility results for three interventions (usual care, education and low cost/free equipment) with a non-zero probability of being the most cost-effective intervention at willingness-to-pay thresholds of less than £100,000 per QALY are presented. The results were mainly sensitive to the baseline incidence of unintentional poisoning and the number of children in a household. The ICER for education compared with usual care decreased to £19,315 (95 % CrI £6049–£54,810) and £18,275 (95 % CrI 5599–£51,842) per QALY gained when the incidence of unintentional poisoning was increased to reflect a high incidence rate among under 5 years from two most socially disadvantaged families (SA9 and SA10) respectively. Increasing the number of children in a household to 1.8 to reflect the average number of children in a UK household also decreased the ICER for education compared with usual care to £22,960 (95 % CrI 11,118–£50,852) per QALY gained.

**Table 8:** Results of sensitivity analysis outlined in Additional file 1 for medicinal poisoning decision model. Only results for the three interventions (usual care, education and low cost equipment) are presented

	Expected QALYs	Expected Costs (£s)	Incremental QALYs	Incremental Costs (£s)	ICER (£s per QALY)	Probability intervention is cost-effective at £30,000/QALY	Probability intervention is cost-effective at £50,000/QALY
SA1: Probability that intervention is effective changed from Posterior to the Predictive distribution of intervention effects and baseline rate							
UC	25060 (25040, 25070)	4169 (2872, 6045)				0.850	0.453
E	25060 (25040, 25070)	5463 (4221, 7319)	0.027 (0.001, 0.059)	1298 (1108, 1498)	47160 (19917, 1361570)	0.150	0.540
FE	25060 (25040, 25070)	6300 (5032, 8163)	0.028 (0.002, 0.06)	2140 (1926, 2360)	74625 (33405, 1498841)	0.000	0.006
SA2: Baseline probability of safe storage changed from 75 % (KCS community controls) to 93 % (Patel et al 2008)							
UC	25060 (25040, 25070)	3158 (2030, 4720)				0.998	0.867
E	25060 (25040, 25070)	4056 (2938, 5599)	0.013 (0.006, 0.023)	898 (824, 957)	71065 (37747, 150605)	0.002	0.133
FE	25060 (25040, 25070)	4302 (3184, 5850)	0.013 (0.006, 0.024)	1139 (1052, 1227)	87285 (46839, 184910)	0.000	0.000
SA3: Baseline probability of safe storage changed from 75 % (KCS community controls) to 50 % (Assumption)							
UC	25060 (25040, 25070)	4903 (3542, 7022)				0.942	0.59
E	25060 (25040, 25070)	6885 (5585, 8921)	0.037 (0.017, 0.074)	1985 (1816, 2121)	53970 (26110, 126315)	0.058	0.41
FE	25060 (25040, 25070)	8567 (7273, 10620)	0.036 (0.014, 0.073)	3671 (3458, 3861)	101700 (48946, 270812)	0.000	0.000
SA4: Probability intervention is accepted changed from 90 to 50 % (Assumption)							
UC	25060 (25040, 25070)	4169 (2872, 6045)				0.979	0.745
E	25060 (25040, 25070)	5227 (3965, 7059)	0.017 (0.008, 0.033)	1061 (970.192, 1130)	62195 (30779, 134700)	0.02	0.254
FE	25060 (25040, 25070)	5693 (4429, 7527)	0.017 (0.009, 0.034)	1526 (1423, 1618)	87356 (43931, 178215)	0.000	0.001
SA5: Proportion admitted changed from 63 % (HSE, 2012) to 83.3 % (Phil Miller, personal communication)							
UC	25060 (25040, 25070)	5140 (3430, 7606)				0.625	0.146
E	25060 (25040, 25070)	6358 (4711, 8776)	0.036 (0.018, 0.066)	1214 (1023, 1372)	33630 (17180, 74539)	0.374	0.852
FE	25060 (25040, 25070)	7202 (5548, 9627)	0.036 (0.018, 0.068)	2043 (1829, 2242)	55495 (28904, 118515)	0.000	0.002
SA6: Provided two pop it locks costing £2.65 per lock.							
UC	25060 (25040, 25070)	4169 (2872, 6045)				0.828	0.298
E	25060 (25040, 25070)	5435 (4197, 7271)	0.031 (0.015, 0.059)	1273 (1110, 1398)	41330 (20007, 91534)	0.17	0.677
FE	25060 (25040, 25070)	5787 (4548, 7596)	0.031 (0.016, 0.061)	1629 (1462, 1772)	51685 (25478, 107910)	0.002	0.026
SA7: Provided two magnetic locks costing £4.80 per lock.							
UC	25060 (25040, 25070)	4169 (2872, 6045)				0.828	0.301
E	25060 (25040, 25070)	5435 (4197, 7271)	0.031 (0.015, 0.059)	1273 (1110, 1398)	41330 (20007, 91534)	0.172	0.698
FE	25060 (25040, 25070)	6751 (5491, 8585)	0.031 (0.016, 0.061)	2592 (2383, 2779)	82570 (41555, 16911)	0.000	0.000
SA8: Increase the number of children in a household from 1 to 1.8							
UC	25060 (25040, 25070)	4169 (2872, 6045)				0.242	0.026
E	25060 (25040, 25070)	5435 (4197, 7271)	0.031 (0.015, 0.059)	1273 (1110, 1398)	22960 (11118, 50852)	0.755	0.962
FE	25060 (25040, 25070)	6270 (5027, 8099)	0.031 (0.016, 0.061)	2111 (1926, 2275)	37210 (18628, 77301)	0.003	0.012
SA9: Change incidence of medically reported poisonings from 30.1 to 44.9 per 10,000 person-years (rate of unintentional poisonings among under 5 year olds in the 4^th^ most deprived quintile, Orton et al 2014 [10])							
UC	25060 (25040, 25070)	5963 (3814, 8986)				0.226	0.040
E	25060 (25040, 25070)	7110 (5119, 10070)	0.06 (0.023, 0.179)	1171 (911, 1355)	19315 (6049, 54810)	0.764	0.929
FE	25060 (25040, 25070)	7958 (5944, 10882)	0.062 (0.024, 0.183)	2002 (1721, 2215)	32024.99 (10358, 85853)	0.010	0.030
SA10: Change incidence of medically reported poisonings from 30.1 to 48.5 per 10,000 person-years (rate of unintentional poisonings among under 5 year olds in the 5^th^ most deprived quintile, Orton et al 2014 [10])							
UC	25060 (25040, 25070)	6380 (4239, 9731)				0.172	0.031
E	25060 (25040, 25070)	7539 (5451, 10751)	0.062 (0.024, 0.182)	1148.5 (879, 1339)	18275 (5599, 51842)	0.818	0.938
FE	25060 (25040, 25070)	8375 (6296, 11590)	0.063 (0.026, 0.189)	1983 (1695, 2208)	30759.974 (9962, 81214)	0.010	0.030
SA11: Change estimate of standard error of utility decrements from 10 to 20 % of mean utility decrement value (assumption)							
UC	25060 (25040, 25070)	4131 (2842, 6011)				0.800	0.800
E	25060 (25040, 25070)	5409 (4187, 7176)	0.031 (0.015, 0.062)	1289 (1125, 1426)	40770 (19540, 92562)	0.200	0.200
FE	25060 (25040, 25070)	6283 (5038, 8041)	0.032 (0.016, 0.064)	2152 (1960, 2332)	66850 (32697, 141502)	0.000	0.000
SA12: Change estimate of standard error of utility decrements from 10 to 50 % of mean utility decrement value (assumption)							
UC	25060 (25040, 25070)	4095 (2899, 6038)				0.794	0.304
E	25060 (25040, 25070)	5382 (4238, 7217)	0.031 (0.014, 0.062)	1290 (1124, 1420)	41265 (19450, 100505)	0.206	0.696
FE	25060 (25040, 25070)	6257 (5082, 8100)	0.032 (0.014, 0.063)	2155 (1966, 2332)	66825 (32717, 160607)	0.000	0.000

### Non-medicinal poisoning results

Results of the base-case decision model investigating the cost-effectiveness of interventions to prevent unintentional poisonings due to ingestion of other household products are presented in Table 9 (cost-effectiveness analysis) and Table 10 (cost-utility analysis). In both analyses, home safety education interventions were associated with more costs but fewer health benefits (i.e. more poison cases in the cost-effectiveness analysis and fewer QALYs in the cost-utility analysis) than usual care. Consequently, all active interventions were dominated by usual care with the ICERs relative to usual care lying in the north-west quadrant of the cost-effectiveness plane. We replicated the sensitivity analyses listed in Additional file 1 for the safe storage of other household products model but none resulted in substantial change to the base case cost-effectiveness results.

**Table 9:** Base case cost-effectiveness results for non-medicinal (other household products) model

Intervention	Expected benefits (Poison cases)^a^	Expected Costs (£)^a^	Incremental benefits (Poison avoided) ^a^	Incremental Costs (£)^a^	ICER (£/Poison avoided)	Probability intervention is cost-effective at £30,000/QALY	Probability intervention is cost-effective at £50,000/QALY
UC	5.820 (4.848, 7.010)	2242 (1563, 3272)				1.000	1.000
E	5.925 (4.862, 7.252)	3544 (2839, 4626)	−0.106 (−0.272, −0.004)	1305 (1231, 1389)	Dominated	0.000	0.000
FE	5.892 (4.855, 7.188)	4650 (3916, 5774)	−0.016 (−0.343, 0.000)	2414 (2202, 2656)	Dominated	0.000	0.000
E + FE	5.949 (4.865, 7.326)	5979 (51644, 7124)	−0.123 (−0.304, −0.005)	3728 (3365, 4128)	Dominated	0.000	0.000
E + FE + HSI	5.954 (4.86, 7.307)	5736 (4933, 6861)	−0.129 (−0.311, −0.006)	3491 (3153, 3867)	Dominated	0.000	0.000
E + FE + F	5.931 (4.862, 7.279)	6291 (5458, 7421)	−0.110 (−0.279, −0.005)	4035 (3622, 4480)	Dominated	0.000	0.000
E + FE + HSI + F	5.879 (4.864, 7.259)	4101 (3390, 5181)	−0.034 (−0.352, 0.000)	1864 (1717, 2040)	Dominated	0.000	0.000

^a^ Firgures are expected QALY (95 % credibility interval) and expected costs (95 % credibility interval) per 1000 households over a lifetime horizon (assumed equal to 100 years)

*Probability CE* probability that intervention is cost effective at a £30,000/£50,000 threshold value. *QALYs* quality-adjusted life years

Interventions

*UC* usual care

*E* education

*FE* provision of low cost/free equipment

*E + FE* education + provision of low cost/free equipment

*E + FE + HSI* education + provision of low cost/free equipment + home safety inspection

*E+ FE + F* education + provision of low cost/free equipment + Fitting

*E + FE + HSI + F+ fitting* education + provision of low cost/free equipment + home safety inspection + Fitting

**Table 10:** Base case cost-utility results for non-medicinal (other household products) decision model

Intervention	Expected benefit (QALY)^a^	Expected Cost (£)^a^	Incremental benefit (QALY)^a^	Incremental Costs (£)^a^	ICER (£/QALY) ^a^	Probability intervention is cost-effective at £30,000/QALY	Probability intervention is cost-effective at £50,000/QALY
UC	25056.593 (25039.908, 25074.762)	2483 (1737, 3582)				1.000	1.000
E	25056.580 (25039.900, 25074.754)	3794 (3022, 4970)	−0.006 (−0.021, 0.000)	1308 (1235, 1403)	Dominated	0.000	0.000
FE	25056.576 (25039.900, 25074.750)	4901 (4109, 6097)	−0.001 (−0.027, 0.000)	2414 (2206, 2665)	Dominated	0.000	0.000
E + FE	25056.578 (25039.898, 25074.754)	6219 (5312, 7432)	−0.007 (−0.025, 0.000)	3733 (3358, 4163)	Dominated	0.000	0.000
E + FE + HSI	25056.578 (25039.898, 25074.752)	5978 (5086, 7180)	−0.007 (−0.026, 0.000)	3497 (3153, 3885)	Dominated	0.000	0.000
E + FE + F	25056.580 (25039.898, 25074.754)	6524 (5611, 7743)	−0.006 (−0.022, 0.000)	4038 (3628, 4507)	Dominated	0.000	0.000
E + FE + HSI + F	25056.576 (25039.900, 25074.750)	4354 (3583, 5490)	−0.001 (−0.027, 0.000)	1864 (1722, 2056)	Dominated	0.000	0.000

^a^ Figures are expected QALY (95 % credibility interval) and expected costs (95 % credibility interval) per 1000 households over a lifetime horizon (assumed equal to 100 years)

*Probability CE* probability that intervention is cost effective at a £30,000/£50,000 threshold value. *QALYs* quality-adjusted life years

Interventions

*UC* usual care

*E* education

*FE* provision of low cost/free equipment

*E + FE* education + provision of low cost/free equipment

*E + FE + HSI* education + provision of low cost/free equipment + home safety inspection

*E+ FE + F* education + provision of low cost/free equipment + Fitting

*E + FE + HSI + F+ fitting* education + provision of low cost/free equipment + home safety inspection + Fitting

## Discussion

### Summary of findings

Education or provision of low cost/free equipment had lower ICERs compared to usual care than more intensive interventions for the prevention of medicinal poisonings. At a cost-effectiveness threshold of £30,000 per QALY gained, education provided to families in disadvantaged areas (£19,315 per QALY gained) or to families with more than one child aged 0–4 years (£22,960 per QALY gained) were the only cost-effective interventions. None of the interventions were found to be cost effective for the prevention of poisoning by non-medicinal substances.

### Strengths and limitations

The evaluations are fully probabilistic, allowing parameter uncertainty to be taken into account in the cost-effectiveness estimates. Where uncertainty remains, for example because of model assumptions or uncertainty about which piece of evidence to use when multiple sources are available for the same parameter, these were investigated through scenario and deterministic sensitivity analyses. The results of these analyses were largely robust to many of the changes in the parameter values we tested. There are two exceptions - restricting the analysis to under 5 year olds from disadvantaged groups and increasing the number of children per household to 1.8 both increased the cost-effectiveness of home safety interventions compared with usual care. To improve transparency in our model, we presented cost-effectiveness estimated in the natural units (number of poison cases avoided) as well as in QALYs. The estimates based on the cost per QALY can help inform prioritisation of healthcare resources, whilst those from the cost per poison case avoided primarily served to validate the model predictions of the likely benefit associated with each intervention and the direction of cost-effectiveness.

The main limitation stems from the lack of high-quality data on the clinical history and prognosis of childhood poisoning, background utility norms and poisoning-related quality of life in children. Data on background utility norms for children were unavailable, so we extrapolated utility norms for the 18–25 year group from Kind et al [21] to earlier age groups in the model. However, preferences for health may differ between children and adults. The impact of ill health on emotional development, education and future prospects may disproportionately affect children, yet this may not be captured by utility norms obtained using instruments and valuation techniques designed for adult populations. Also, variations in utility weights across different countries have been reported for conditions such strokes [25] and between self- and proxy-reported measures of health-related quality of life in children aged 5–14 years [26]. Hence it is possible that the utility decrements used in our model may not adequately reflect preferences for a UK injured population as they were obtained from two American studies and proxy elicited by clinicians [22, 23].

Our costing assumed poison prevention education and a home safety inspection each required 5 min of health visitor time, and fitting equipment required 5 min of equipment fitter time. Safety equipment schemes usually provide advice and equipment aimed at preventing a range of injuries, not just poisonings and it is possible that we overestimated the cost of poison prevention interventions. Furthermore, our analyses do not take account of potential co-benefits from other prevention advice or equipment, for example, providing safety gates to prevent access to kitchens where hazardous substances may be stored. It is therefore possible that our study underestimates the cost-effectiveness of poison prevention interventions.

Although we found that providing education was the only cost-effective intervention, it is important to remember that education alone is not the most effective poison prevention intervention [9], that there are steep social gradients in childhood poisoning [10] and that disadvantaged families often cannot afford safety equipment [27]. Reducing inequalities in childhood poisoning is an important policy objective, and our analyses did not address this issue.

Our findings are unlikely to be generalizable to countries, such as the USA, with poison control centres. These centres offer a round the clock service for the general public, providing free information and advice following a poisoning and also on poison prevention. They aim to reduce hospital visits by providing treatment at home. This is likely to result in substantial differences in healthcare resource use in countries with and without such services.

Whilst acknowledging the limitations in our adaptation of the QALY framework for evaluation of poison prevention interventions in children, our primary motivation was to produce cost-effectiveness estimates to inform prioritisation of scarce public health resources. In the UK, this requires that cost-effectiveness is expressed in QALYs [12, 28]. Even when QALYs are estimable in our analysis, they may not necessarily capture all benefits of a public health intervention. For example, parents and the economy may benefit from not having to take time off work to care for injured children, but a lack of data precluded the inclusion of this benefit in the model. Other externalities not captured by the perspective adopted in our analysis include non-health gains to other children and the emotional distress avoided of not losing a child due to poisoning for parents, grandparents and siblings. Our cost-effectiveness estimates should therefore be treated as conservative as the true benefit of the interventions may have been underestimated.

### Comparisons with previous work

A previous economic evaluation of strategies for preventing unintentional injuries in children is the analysis undertaken to inform the development of NICE PH30 [Preventing unintentional injuries among under-15 s in the home] [13]. Pitt et al. evaluated a generic home safety intervention versus no intervention for the prevention of any injury in the home irrespective of injury mechanism (i.e. injury included falls, scalds, poisonings, etc.). Their analysis found home safety interventions have an ICER of £187,154 per QALY gained compared with usual care intervention. The analysis presented here builds on Pitt et al.*’s* by evaluating the cost-effectiveness of several interventions (all of which are more homogenously defined than the strategy in Pitt et al.) to prevent medicinal and non-medicinal poisons in pre-school children.

### Recommendation for research

The valuation of health related quality of life in children is an active area of current research but progress is hampered by the fact that children, especially those under 5 years old, usually lack the cognitive ability and emotional maturity required to provide appropriate responses to quality of life questionnaires. A number of generic health utility instruments such Paediatric Quality of Life Inventory (PedsQL) [29, 30] and child versions of the adult EuroQoL five-dimensional (EQ-5D) questionnaire [31] are available for use in studies of child health. Currently the use of these instruments to inform economic evaluation studies of child interventions is restricted by lack of research to derive the relevant utility weights. Future research should be directed at developing strategies for eliciting the children health preferences that can be used to generate utility weights and health related quality of life information in children. In the meantime, a promising area of research in the absence of child related utility weights is the development of algorithms to derive coefficients that can be used to map the scores from an instrument where utility weights are not available to an instrument where the utility weights are available. Khan et al. [32] derived such coefficients for converting PedsQL scores to EQ-5D scores using data from a cross-sectional survey of English school children aged 11–15 years. These type of mapping exercises should be conducted on data on different clinical conditions and different age groups to assess their usefulness in health technology assessment and public health evaluations. As poison prevention interventions are often provided as part of interventions aimed at preventing a range of injuries (such as safety equipment schemes or home safety inspections), more complex decision models are needed to evaluate costs and effectiveness across a range of interventions and outcomes in a single analytic model.

### Recommendations for practice

Interventions that provide equipment, plus home safety inspections and fitting of equipment are more effective than education alone. Children from disadvantaged families are at higher risk of poisoning and these families face multiple barriers, including financial barriers to making their homes safer. Reducing inequalities in child poisoning is an important policy objective, but our analyses were not able to address this. Commissioners of home safety interventions therefore need to consider our cost-effectiveness evidence as only one element in their decision making.

## Conclusion

Education offers better value for money than the more intensive but expensive strategies for preventing medicinal poisonings, but is only likely to be cost-effective (at £30,000 per QALY gained) for families in disadvantaged areas or for those with more than one child. None of the interventions were cost effective for preventing non-medicinal poisonings. There was considerable uncertainty in cost-effectiveness estimates due to paucity of evidence on model parameters. Policy makers should consider both the costs and effectiveness of competing interventions to ensure efficient use of scarce resources.

## Abbreviations

DALY, disability adjusted life year; ED, Emergency Department; EQ-5D, EuroQoL 5 dimensional quality of life questionnaire; GBD, global burden of disease; GP, general practitioner; ICER, incremental cost-effectiveness ratio; NHS, National Health Service; NHS/PSS, UK National Health Service and Personal Social Services; NICE, National Institute for Care excellence; NIHR, National Institute for Health Research; PedsQL, paediatric quality of life inventory; PH, public health; PSSRU, personal social services research unit; QALY, quality adjusted life year; UK, United Kingdom; USA, United States of America

## Additional files

Additional file 1: Key assumptions. (DOCX 12 kb) Additional file 2: Sensitivity Analyses (SA). (DOCX 13 kb)

## Footnotes

1 The base case analysis models representative sample of the UK households (i.e. includes a mixture of deprived and non-deprived households).

2 We assumed a single child per household in the base case analysis.
