# Supplementary material for: A decision analytic model to investigate the cost-effectiveness of poisoning prevention practices in households with young children
Source: BMC Public Health. 2016 Aug 3;16:705. doi: 10.1186/s12889-016-3334-0 (PMC4973049; doi:10.1186/s12889-016-3334-0)
Supplement: Additional file 1: — Key assumptions. (DOCX 12 kb) [file 12889_2016_3334_MOESM1_ESM.docx]

| **Additional file 1: key assumptions** | |
| --- | --- |
| 1 | Households are assumed to have one child under 5 years old |
| 2 | Actual or suspected medical case of reported unintentional ingestion of a poison is taken to the emergency department for assessment and or treatment. Cases triaged as minor are treated and discharged from the emergency department. Cases assessed as moderate are assumed to be admitted to hospital for short in-patient stay. Severe poisoning including fatalities are assumed to be admitted for a period of long-inpatient stay. Unintentional poisoning after 5 years of age are excluded in the model. Severe injuries lead to chronic injury/ health condition that persists for the rest of the individual life. |
| 3 | Acceptance rate of the intervention among the modelled population is 90% |
| 4 | Uncertainty associated with utility decrement as result of poison injury assumed to be 10% of the mean utility decrement |
| 5 | Assumptions made in estimating the resource utilisation associated delivery of home safety interventions are described in Table 4 |
