# Supplementary material for: A decision analytic model to investigate the cost-effectiveness of poisoning prevention practices in households with young children
Source: BMC Public Health. 2016 Aug 3;16:705. doi: 10.1186/s12889-016-3334-0 (PMC4973049; doi:10.1186/s12889-016-3334-0)
Supplement: Additional file 2: — Sensitivity Analyses (SA). (DOCX 13 kb) [file 12889_2016_3334_MOESM2_ESM.docx]

| **Additional file 2: Sensitivity Analyses (SA)** | |
| --- | --- |
| SA1 | Estimate the probability that the intervention is effective from the predictive distribution of the relative treatment effects. The predictive distribution of effects is provides a more conservative estimate of the uncertainty around the relative intervention effects as it incorporates both the uncertainty about the value of a new observation as well as the observed variation in the data in both the relative effects and absolute effects under usual care intervention. |
| SA2 | Baseline probability of safe storage changed from 75% (KCS community controls) to 93% (Patel et al 2008) |
| SA3 | Baseline probability of safe storage changed from 75% (KCS community controls) to 50% (Assumption) |
| SA4 | Probability intervention is accepted changed from 90% to 50% (Assumption) |
| SA5 | Proportion admitted changed from 63% (HSE, 2012) to 83.3% (Phil Miller, personal communication) |
| SA6 | Provided two pop-in locks costing £2.65 each. |
| SA7 | Provided two magnetic locks costing £4.80 per lock. |
| SA8 | Increased the number of children in a household from 1 to 1.8. |
| SA9 | Changed incidence of unintentional poisoning injury from 18.07 per 10,000 person-years (medicinal poisonings) to 44.9 per 10,000 person-years for the households with socio-economic deprivation index of 4 [[9](#_ENREF_9)]. |
| SA10 | Changed incidence of unintentional poisoning injury from 18.07 per 10,000 person-years (medicinal poisonings) to 48.5 per 10,000 person-years for households with socio-economic deprivation of 5 (i.e. most socio-economically deprived group [[9](#_ENREF_9)]). |
| SA11 | Changed estimate of standard error of utility decrements from 10% to 20% of mean utility decrement value (assumption) |
| SA12 | Changed estimate of standard error of utility decrements from 10% to 50% of mean utility decrement value (assumption) |
